# Editorial: Amplifying the voices of individuals with visual impairments and deaf-blindness in the context of sports

**DOI:** 10.3389/fspor.2025.1750098

**Published:** 2025-12-11

**Authors:** Giese Martin, Meier Stefan, Höger Brigitta

**Affiliations:** 1 Department of Education, University of Marburg, Marburg, Germany; 2 Faculty of Philosophy and Social Sciences, Universitat Augsburg, Augsburg, Germany

**Keywords:** inclusion, visual impairment, blindness, deaf-blind, physical activity

The global inclusion movement and the United Nations Convention on the Rights of Persons with Disabilities (CRPD) stress the urgent need to address ongoing marginalization and discrimination against people with disabilities. Despite some progress, individuals with visual impairments and deaf-blindness still face significant barriers to participating in sports, physical activity, and physical education. Research continues to reveal gaps in our understanding of their perspectives and experiences—insights that are crucial for building truly inclusive environments. To close these gaps, it is essential to center and elevate the voices of people with visual impairments and deaf-blindness within academic discussions. Accordingly, this research topic aims to explore the challenges faced and insights shared by people with blindness, visual impairment (BVI), and deaf-blindness, providing a deeper understanding of their experiences and helping to transform society. By identifying current obstacles and suggesting practical solutions, this work seeks to encourage greater participation and inclusion for individuals with BVI and deaf-blindness in these areas.

In order to paint as broad and diverse a picture as possible of the barriers to participation, as many perspectives as possible are considered. For example Höger et al. studied the social hierarchies in segregated physical education (PE) classes for blind and visually impaired (BVI) students in Austria. Using Clark's Mosaic Approach with school tours and interviews of 19 BVI students and three sighted PE teachers, they identified three main forms of hierarchy: differences between sighted and BVI students supporting segregation, distinctions among BVI students based on their vision level affecting teaching and student views, and varying perceptions of students’ development by teachers vs. students themselves. The study found that while inclusion is possible by challenging ableist norms, these biases often persist subtly.

Markov-Glazer et al. team interviewed 23 Deaflympic athletes and four coaches to explore attitudes toward mental training. They identified three themes: sport psychology consultation, visual orientation in psychological skills, and how Deaf sport culture affects communication. Interest in sport psychology was high, but barriers like limited sign language consultants and structural challenges hindered access. Deaf athletes adapted strategies to suit their visual strengths, facing unique advantages and obstacles. Communication styles varied between native signers and spoken-language users. The study highlights the need for culturally sensitive sport psychology and equitable access for Deaf athletes.

Barrera-Garcimartín et al. presented a case study of a Spanish blind judo Paralympian, covering her journey from Athens 2004 to Paris 2024 through interviews and written accounts. The study identified two critical phases in disabled athletes’ careers: starting sports and preparing for Paralympic participation. It notes barriers faced by women with disabilities, the role of support systems, and the positive impact of initiatives like Paralympic School Days. The athlete emphasized how rules, sport type, and inclusion affect talent development, urging increased support for women with disabilities in sport.

Carretti et al. studied adapted sports’ social and educational effects via the first female blind baseball match, surveying 33 women involved. They found no significant differences in wellbeing between visually impaired and sighted participants, highlighting adapted sport's empowering and integrative benefits. The research, tied to gender violence awareness, suggests such events foster integration and offer educational approaches to combat discrimination.

Bödicker and Elisath presented the experiences of a 15-year-old visually impaired student who transitioned from mainstream inclusive schools to a special school. The study, based on a semi-narrative guided interview, explores the interplay between empowering personal and non-personal factors and participation in sports and physical education contexts. The findings critically reflect on the teachers’ role in either facilitating or hindering participation, emphasizing the necessity for teacher education programs that are sensitive to inclusion. The research adopts a critical perspective on ableism, re-examining societal attributions of abilities and highlighting the importance of resilience and vulnerability in sports participation among young people with visual impairments.

Sträter et al. examined how a seminar on blind and visually impaired (BVI) ski guiding affected physical education teacher education (PETE) students’ attitudes toward diversity. Through simulated and real BVI skiing experiences, students gained direct guiding practice. Interviews showed that these experiences improved attitudes, reduced uncertainty, and boosted self-efficacy, though some uncertainty remained due to individual differences. The study underscores the importance of interactive teaching methods for fostering inclusion.

Greve et al. evaluated the BAT-Sailing project, a collaboration between Norddeutscher Regatta Verein and FC St. Pauli Segeln to promote inclusive sailing for athletes with and without disabilities. „BAT“ references bats’ ability to navigate without sight. The project (2021–2023) used Patton's utilization-focused evaluation and found that communication between sighted and blind sailors was vital for teamwork and cooperation. This approach effectively improved inclusive sailing teams and offered insights for future sports initiatives.

Giese and Grenier investigated the barriers faced by blind and visually impaired students in general physical education (PE) classes. Interviewing ten students aged 17–19 who moved from mainstream to specialized schools, they found that PE was particularly challenging and often led to negative experiences for these students. The study emphasizes the need to review exclusionary practices in PE and promote inclusion by directly considering the perspectives of visually impaired students.

Oldörp et al. explored how different abilities influence inclusive experiences for people with visual impairments in alpine skiing. Through interviews with six German skiers, they found four key themes: skiing boosts self-confidence, serves as proof of skill, allows participants to ski without drawing attention, and provides a way toward greater accessibility and inclusion. The skiers noted improvements in confidence, motor skills, social abilities, and advocacy for better access. Despite viewing skiing as inclusive, they faced obstacles like a shortage of guides, limited awareness of para-skiing, and separate competitions. The research highlights the need to raise awareness, enhance access to guiding, and introduce policy changes so that people with disabilities have equal opportunities, aligning with the UN Convention on the Rights of Persons with Disabilities.

